# Ante-Mortem Invasion by NonPathogenic Microbes

**Published:** 1901-06-01

**Authors:** 


### ANTE-MORTEM INVASION BY NON-PATHOGENIC MICROBES.

The human body while alive is the natural home of the pathogenic microbe. When dead it is taken possession of by others of many kinds, by the action of which it is rapidly decomposed. It is interesting, however, to note that even before death takes place micro-organisms of decomposition may obtain a footing in the body, and that in some cases the final cessation of life is hastened by the intrusion of non-pathogenic organisms which have taken advantage of the diminishing vital resistance of the tissues to implant themselves in their midst. At the last meeting of the Pathological Society, which was held in the physiological laboratory of Guy's Hospital, Dr. J. H. Bryant reported the results of a bacteriological examination of three cases of ante-mortem thrombi. In the first case, which was one of mitral stenosis and tricuspid regurgitation, extensive ante-mortem thrombosis was found in which streptococci and staphylococcus albus were discovered. In the second case a pure culture of the proteus vulgaris was obtained, but the examination was not made until twenty hours after death. In the third a pure culture of the bacillus subtilis was obtained from the thrombus in the left saphenous vein and of the staphylococcus albus from the vein on the right side. This post-mortem was made four hours after death. It seemed possible that in the last case the bacillus subtilis had found its way into the blood current through an old ulcer the patient had on his leg.

